# Spatial distribution and insecticide resistance profile of *Aedes aegypti* and *Aedes albopictus* in Douala, the most important city of Cameroon

**DOI:** 10.1371/journal.pone.0278779

**Published:** 2022-12-13

**Authors:** Aurelie P. Yougang, Christophe R. Keumeni, Theodel A. Wilson-Bahun, Armel N. Tedjou, Flobert Njiokou, Charles Wondji, Basile Kamgang

**Affiliations:** 1 Centre for Research in Infectious Diseases, Yaoundé, Cameroon; 2 Faculty of Science, Parasitology and Ecology Laboratory, Department of Animal Biology and Physiology, University of Yaoundé 1, Yaoundé, Cameroon; 3 Faculty of Science and Technology, Laboratory of Vertebrate and Invertebrate Bioecology, Marien-Ngouabi University, Brazzaville, Congo; 4 Vector Biology Department, Liverpool School of Tropical Medicine, Liverpool, United Kingdom; Fundacao Oswaldo Cruz Instituto Rene Rachou, BRAZIL

## Abstract

Prevention and control of *Aedes*-borne viral diseases such as dengue rely on vector control, including the use of insecticides and reduction of larval sources. However, this is threatened by the emergence of insecticide resistance. This study aimed to update the spatial distribution, the insecticide resistance profile of *A*. *aegypti and A*. *albopictus* and the potential resistant mechanisms implicated in the city of Douala. Immature stages of *Aedes* were collected in August 2020 in eight neighbourhoods in Douala and reared to adult stages. Adult bioassays, and piperonyl butoxide (PBO) synergist assays were carried out according to World Health Organization recommendations. Expression of some candidate metabolic genes including *Cyp9M6F88/87*, *Cyp9J28a*, *Cyp9J10* and *Cyp9J32* in *A*. *aegypti*, and *Cyp6P12* in *A*. *albopictus* were assessed using qPCR. *A*. *aegypti* adults G0 were screened using real time melting curve qPCR analyses to genotype the F1534C, V1016I and V410L *Aedes kdr* mutations. Overall, *A*. *aegypti* is the predominant *Aedes* species, but analyses revealed that both *A*. *albopictus* and *A*. *aegypti* coexist in all the prospected neighbourhoods of Douala. High level of resistance was observed to three pyrethroids tested in both *Aedes* species. In *A*. *aegypti* a lower mortality rate was reported to permethrin (5.83%) and a higher mortality rate to deltamethrin (63.74%). Meanwhile, for *A*. *albopictus*, lower (6.72%) and higher (84.11%) mortality rates were reported to deltamethrin. Similar analysis with bendiocarb, revealed for *A*. *aegypti* a loss of susceptibility. However, in *A*. *albopictus* samples, analyses revealed a susceptibility in Logbessou, and confirmed resistance in Kotto (59.78%). A partial recovery of mortality was found to insecticides after pre-exposure to PBO. *Cyp6P12* was found significantly overexpressed in *A*. *albopictus* permethrin resistant and *Cyp9M6F88/87* for *A*. *aegypti* deltamethrin resistant. F1534C, V1016I and V410L mutations were detected in *A*. *aegypti* from different neighbourhoods and by considering the combination of these three kdr 14 genotypes were found. These findings provide relevant information which should be capitalised in the implementation of arbovirus vector control strategies and insecticide resistance management.

## Introduction

*Aedes*-borne viral diseases such as dengue, chikungunya, yellow fever and Zika are increasingly reported in Cameroon. Recently it was demonstrated that 13% of acute febrile patients consulting hospitals in Douala, Cameroon are due to dengue. During this study the co-circulation of three dengue serotypes, DENV-1, DENV-2, and DENV-3, were reported [1]. The viruses responsible for these diseases are transmitted by the infected bite of mosquitoes of the genus *Aedes*, whose major vectors are *Aedes aegypti* Linnaeus, 1762 and *Aedes albopictus* (Skuse), 1894. *Aedes aegypti* and *A*. *albopictus* are from different origins. *A*. *aegypti* originates from Africa [2] and is found throughout the tropics, while *A*. *albopictus* is native to Asia [3], but has invaded all the continents including Africa [4]. Both *Aedes* species are present in Cameroon with discrepancy in distribution. *A*. *aegypti* is distributed across the country while *A*. *albopictus* is restricted to the southern part of the country in latitude 6°N where it tends to supplant the native species *A*. *aegypti* [5–7]. In cities where both species are found, *A*. *aegypti* is found most prevalent in downtown neighbourhoods with high building density while *A*. *albopictus* is mostly found in peri-urban neighbourhoods surrounded by vegetation [5,8–10].

In absence of effective vaccine and specific treatment against this arboviruses, dengue fever prevention relies on vector control through reduction of larval sources and environment management, but during an epidemic, space spraying of insecticides is generally used to target adult mosquitoes [11]. Unfortunately, vector control is counteracted by the emergence and the continued development of resistance to all classes of insecticides used in public health [12].

Two main mechanisms are implicated in *Aedes* mosquitoes’ resistance to insecticides: the metabolic resistance and the target site resistance. Metabolic resistance is mediated by upregulation of detoxification enzymes such as the monooxygenases (cytochrome P450s), glutathione S-transferases (GSTs) and carboxylesterases (COEs) [13]. Many genes of the P450 family, especially from the CYP9 and CYP6 subfamilies (*CYP9J28*, *CYP9J10*, *CYP9J26*, *CYP6BB2* and *CYP6P12*) have been associated with resistance to pyrethroids [12,14–18]. Target site resistance is caused by mutations in target genes such as the acetylcholinesterase (*Ace-1*), the *GABA* receptor and the voltage-gated sodium channel (*VGSC*) which causes knockdown resistance (*kdr*). The *kdr* is one of the main target site resistance mechanisms known as involved in the resistance for both pyrethroids and dichlorodiphenyltrichloroethane (DDT) insecticides [19–21]. In *A*. *aegypti*, 11 *kdr* mutations at 9 different codons positions in the VGSC domains I-IV have been reported [12,22]. Among them F1534C, V410L and V1016G, have already been identified in Cameroon [23–25]. Till date, only four VGSC mutations have been detected in *A*. *albopictus* affecting two codons (1532 and 1534). This study presents the spatial distribution and the insecticide susceptibility profile of *A*. *aegypti* and *A*. *albopictus* and the potential mechanisms implicated in the resistance of these arbovirus vectors in the city of Douala.

## Material and methods

### Mosquito collection and rearing

**Fig 1:**
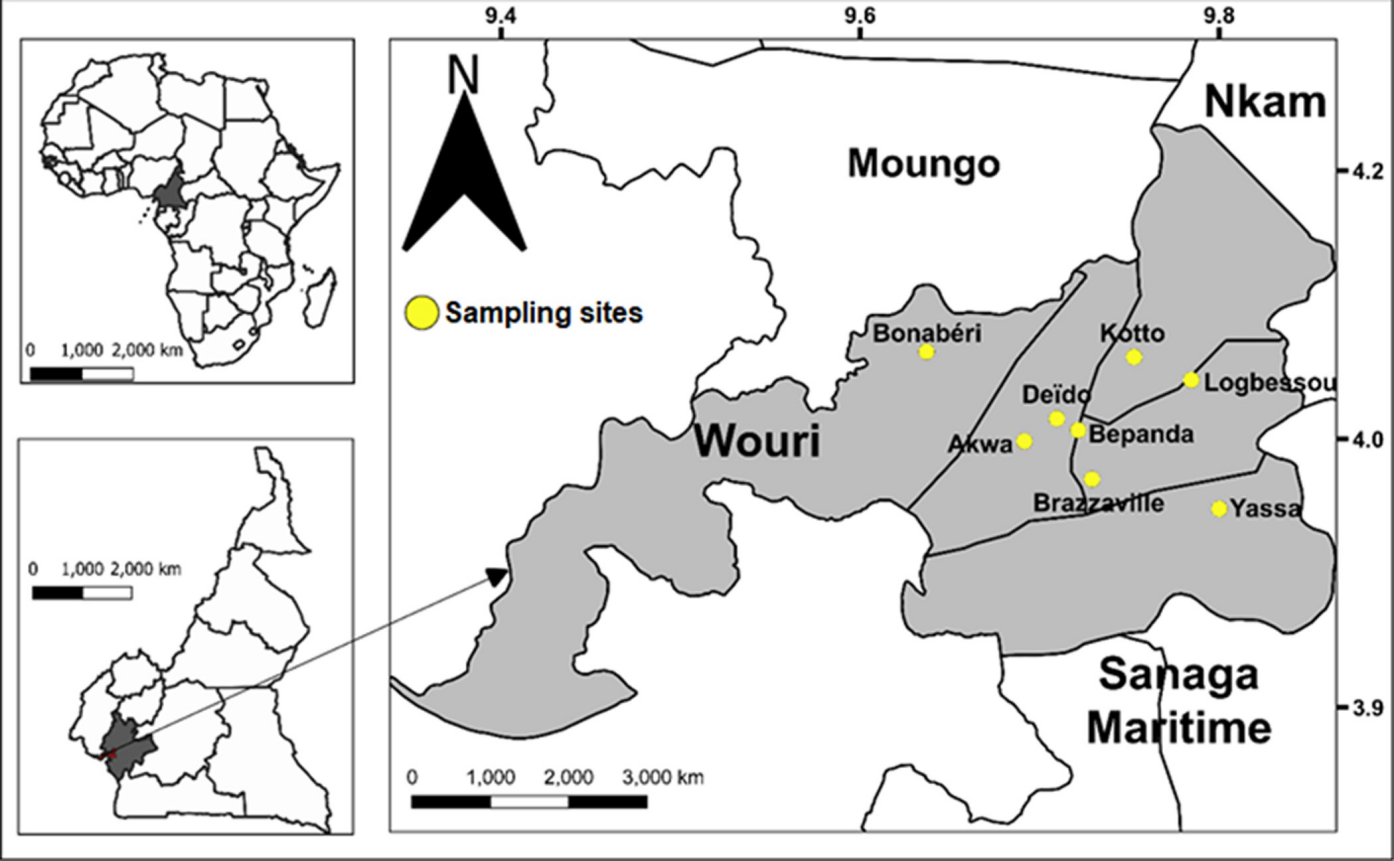
Map of study site. QGIS version 3.14.16, was used to generate the map using open access share files (https://gadm.org/).

*Aedes* mosquitoes were sampled as larvae or pupae in August 2020 (small rainy season) in eight neighbourhoods in Douala (Fig 1). Based on previous studies in Cameroon showing that *A*. *albopictus* is prevalent in neighbourhoods located in suburban areas of the city while A. *aegypti* is predominant in the downtown [6,9], we selected four urban neighbourhoods: central Akwa (N 04°02.934’; E 009°41.475’), Bépanda (N 04°03.303’; E 009°43.272’), Brazzaville (N 04°1.6570’; E 009°43.7310’) and Deïdo (N 04°03.690’; E 009°42.560’) and four peri-urban neighbourhoods: Bonabéri (N 04°04.214’; E 009°41.155’), Kotto (N 04°05.753’; E 009°45.141’), Logbessou (N 04°04.983’; E 009°47.050’) and Yassa (N 04°00.661’; E 009°47.983’). Immature stages (field generation, G0) were collected from different breeding sites: domestic (e.g. tanks), peri-domestic (e.g. used tires, discarded tanks), and natural (e.g. tree holes). In each location, larvae or pupae from at least 20 positive larval breeding places were collected, stored in plastic boxes, and transferred to the insectary, pooled according to the neighbourhoods, and reared to adult stages. Adult mosquitoes were morphologically identified using taxonomic keys [26], numbered, pooled in a breeding cage according to species and location and further reared in controlled conditions (27 ± 2°C; relative humidity 80 ± 10%) until generation 1 (G1) for adult bioassays. The comparison between the prevalence of *A*. *aegypti* and *A*. *albopictus* in each neighbourhood was performed using chi-square tests.

### Insecticide resistance bioassays

Bioassays were performed according to WHO protocol using 2–5 days old G1 generation. Four replicates of 20–25 females per tube were exposed to 0.03% deltamethrin, 0.05% alphacypermethrin, 0.1% bendiocarb and 0.75% permethrin for 1hour. Mortality was recorded 24 hours later and mosquitoes alive or dead after exposure were stored in RNA later or silica gel, respectively. The resistance status was defined as follows: susceptible (mortality rate between 98–100%), probable resistance (mortality rate between 90–97%), and resistant (mortality rate inferior to 90%) [27].

### Adult synergist assay with PBO

To evaluate the potential role of oxidase specific metabolic resistance mechanisms, synergist assays with 4% piperonyl butoxide (PBO) were performed. 2-5-days-old adults were pre-exposed for one hour to PBO-impregnated papers and after that immediately exposed to the selected insecticide. Mortality was scored 24 hrs later and compared to the results obtained with each insecticide without synergist according to the WHO standards [27]. The comparison of mortality rates after pre-exposure of mosquitoes to synergist and without pre-exposure to synergist was done using Chi-square test. The difference was statistically different when *P-value* was inferior to 0.05.

### Knockdown resistance (kdr) genotyping

Three *kdr* mutations (V1016I, V410L and F1534C) were genotyped using around 30 samples per neighbourhood. Genomic DNA extracted from 30 individual mosquitoes per populations [28] were used for this experiment. Genotyping of the V1016I, V410L and F1534C mutations was performed by the real-time quantitative PCR using protocol published by Saavedra-Rodriguez et al.[29,30]. Each PCR reaction was performed in a 21.5 μl mixture containing 2 μl of DNA sample, 10 μl of SYBR® Green (SuperMix), 1.25 μl of each primer and 5.75 μL of ddH2O. The thermocycle parameters were: 95°C for 3 min, followed by 40 cycles of 95°C for 20 s, 60°C for 1min and 72°C for 30 s and then a final step of 72°C for 5 mins, 95°C for 1 min, 55°C for 30 s and 95°C for 30 s.

### Expression of detoxification Genes

#### RNA extraction and cDNA synthesis

For this experiment three groups of mosquitoes were used: the unexposed (control), the exposed (resistant) and the susceptible (laboratory susceptible strains). For each group three replicates of 10 mosquitoes per species were set up. RNA was extracted using the PicoPure RNA Isolation Kit (Arcturus® Picopure RNA Extraction Kit Life Technologies, California, USA), following the manufacturer’s recommendations. Quality and quantity of RNA obtained were assessed using a "NanoDrop Lite" spectrophotometer (Thermo Scientific Inc., Wilmington, USA) and was stored at -80˚C until further use.

Extracted RNA was used to synthesise complementary DNA (cDNA) using the Superscript III kit (Invitrogen, Carlsbad, CA, USA) according to the manufacturer’s instructions, and the resulting cDNA was purified using a QIAquick spin column (QIAuick PCR Purification Kit, Qiagen) and diluted 2-fold to accommodate the volumes of the reaction.

#### Quantitative-reverse transcriptase PCR

Expression profiles of genes previously associated with *A*. *aegypti* or *A*. *albopictus* pyrethroid resistance [12,31] were assessed using quantitative reverse transcription PCR (qRT-PCR), in relation to two susceptible strains, Benin strain for *A*. *aegypti* [32,33] and Vector Control Research Unit (VCRU) strain for *A*. *albopictus* [34,35]. Standard curve analyses were performed for each primer pair to check the specificity and efficiency of amplifications. Four cytochrome P450 candidate genes were chosen for analysis in *A*. *aegypti* (*Cyp9M6F88/87*, *Cyp9J28a*, *Cyp9J10* and *Cyp9J32*) and only one in *A*. *albopictus* (*Cyp6P12*). The reactions were performed in a mixture of 20 μL with 10 μL sybrGreen (Applied Biosystems, Texas, USA), 0.6 μL of each primer (10 μM), 7.8 μL of ddH2O and 1 μL of CDNA, under the following conditions: 95°C for 3 min, followed by 40 cycles of 95°C for 10 s and 60°C for 10 s. The relative expression level and fold change (FC) of each candidate gene compared to susceptible strains were calculated using the 2-ΔΔCT method integrating the efficiency of PCR [36] after normalization with housekeeping genes: *Aaeg60sL8*, *RPS3*, *RSP7* and *qTubulin* (S1 Table). All the primer sequences are presented in Table 1 respectively for *A*. *albopictus* and *A*. *aegypti*. The Mx Pro software integrated into the Agilent brand TaqMan machine was used.

**Table 1:** Primer sequences used to evaluate the level of expression of metabolic resistance genes by RT-qPCR in *Aedes albopictus and Aedes aegypti*.

Genes	Forward primer	Reverse primer
***Aedes albopictus***		
*Cyp6P12*	CGTGCGCTTTTGGGATTGAG	ATCGTCCGTGCCAAATCCTT
*RSP7*	AAGGTCGACACCTTCACGTC	CGCGCGCTCACTTATTAGAT
q*Tubulin*	CCGCACTCGAGAAGGATTAC	GTGGTTCGGTTTGACTTCGT
***Aedes aegypti***		
*Cyp9J10*	ATCGGTGTTGGTGAAAGTTCTGT	CATGTCGTTGCGCATTATCCC
*Cyp9J28*	CCACTGACGTACGATGCGA	GCCGATCAGTGGACGGAGC
*Cyp9M6*	TCGGTGCACAATCCAAACAAC	GTCGGGTACGACCAACGAAA
*Cyp9J32*	CGGTCCGCTTATGACGAAGAG	TTTGTTCGCTCCGAAGAGTGG
*RPS3*	AGCGTGCCAAGTCGATGAA	GTGGCCGTGTCGACGTACT
*Ae60sL8*	CTGAAGGGAACCGTCAAGCAA	TCGGCGGCAATGAACAACT

## Results

### Relative abundance and spatial distribution of *A*. *aegypti* and *A*. *albopictus* in Douala

**Fig 2:**
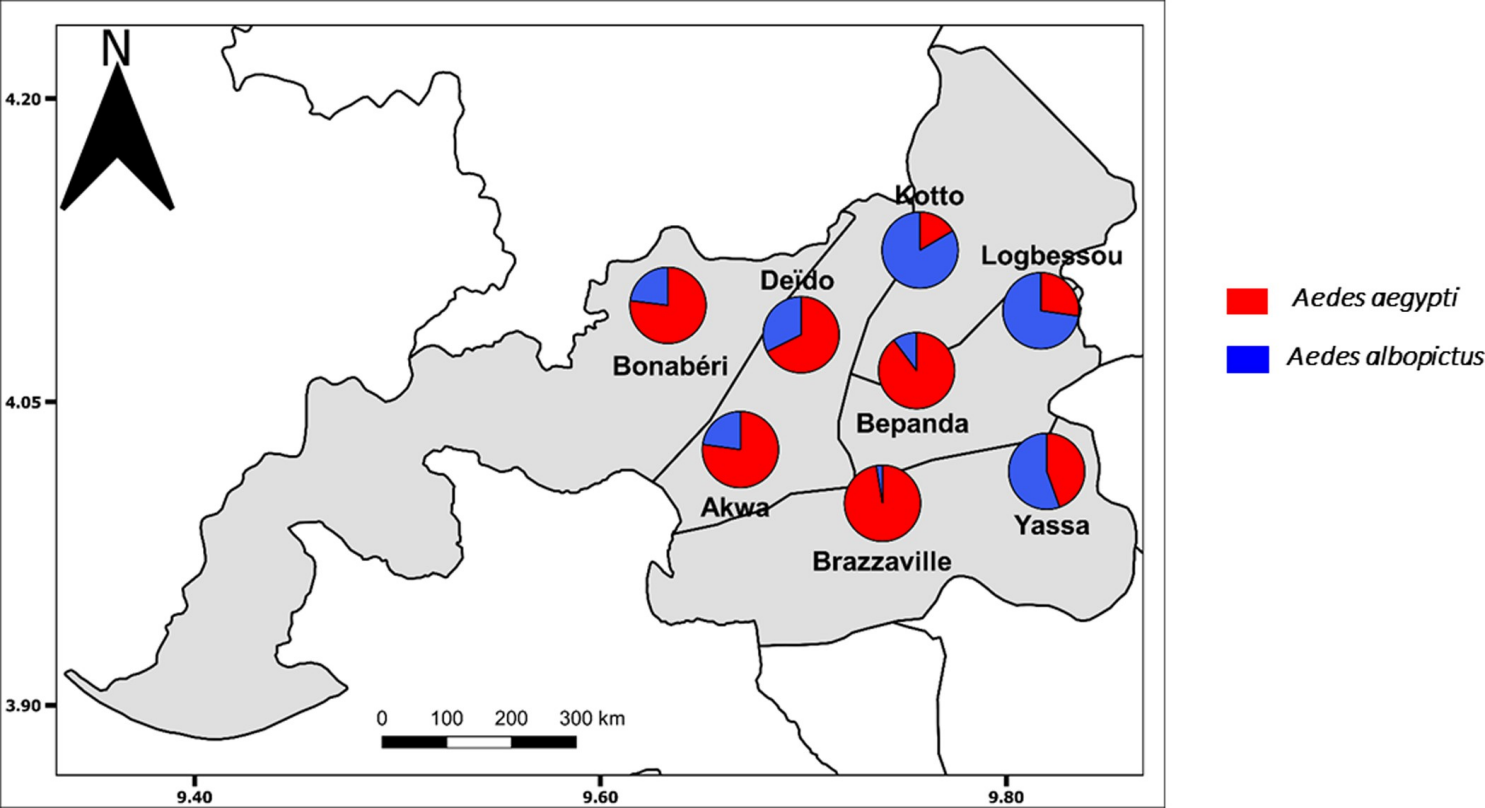
Geographical distribution of *A*. *aegypti* and *A*. *albopictus* in the city of Douala, Cameroon.

A total of 13,927 specimens of *Aedes* spp. comprising 8,564 (61.49%) individuals belonging to *A*. *aegypti* species and 5,363 (38.51%) to *A*. *albopictus* species (Table 2) were collected during this study. Analysis revealed that both *Aedes* species coexist in all the prospected neighbourhoods in Douala but overall, *A*. *aegypti* is the predominant species (Fig 2). According to the environment (downtown vs suburban), *A*. *aegypti* is more predominant in all the neighbourhoods located in the downtown environment (Akwa, Brazzaville, Bépanda and Deïdo) while *A*. *albopictus* is rather predominant in suburban neighbourhoods (Logbessou, Kotto and Yassa) except in Bonabéri where *A*. *aegypti* largely dominated over *A*. *albopictus* (χ² = 2758.8; *P* < 0.001).

**Table 2:** Abundance of *A*. *aegypti* and *A*. *albopictus* from different locations in Douala.

Environment	Location	Breeding sites	Number of prospected breeding sites	Number of positive breeding sites	*A*. *aegypti*	*A*. *albopictus*	*P-value*
**Downtown**	**Akwa**	Used tires	38	25	778	230	< 0.001
		Discarded tanks	3	2			
		Car wrecks	1	1			
	**Bépanda**	Used tires	60	37	1592	182	< 0.001
		Car wrecks	1	1			
	**Brazzaville**	Used tires	104	56	1865	51	< 0.001
	**Deïdo**	Used tires	38	22	973	465	< 0.001
		Factory molds	50	30			
	**Total 1**		**295**	**174**	**5208**	**928**	< 0.001
**Suburban**	**Bonabéri**	Used tires	69	58	1681	503	< 0.001
		Discarded tanks	7	7			
	**Kotto**	Used tires	84	37	291	1471	< 0.001
		Discarded tanks	2	1			
		Miscellaneous	1	1			
	**Logbessou**	Used tires	141	82	516	1378	< 0.001
	**Yassa**	Used tires	252	129	868	1083	< 0.001
	**Total 2**		**556**	**315**	**3356**	**4435**	< 0.001
	**Total 1+Total 2**		**851**	**489**	**8564**	**5363**	**< 0.001**

### Insecticide resistance profile of *A*. *aegypti* and *A*. *albopictus* in Douala

The results of adult bioassays showed a level of susceptibility varying according to the neighbourhood and the insecticide used. Three pyrethroids (0.03% deltamethrin, 0.75% permethrin and 0.05% alphacypermethrin) and one carbamate (0.1% bendiocarb) were used for adult bioassays.

#### Insecticide resistance profile of *A*. *aegypti* in Douala

**Fig 3:**
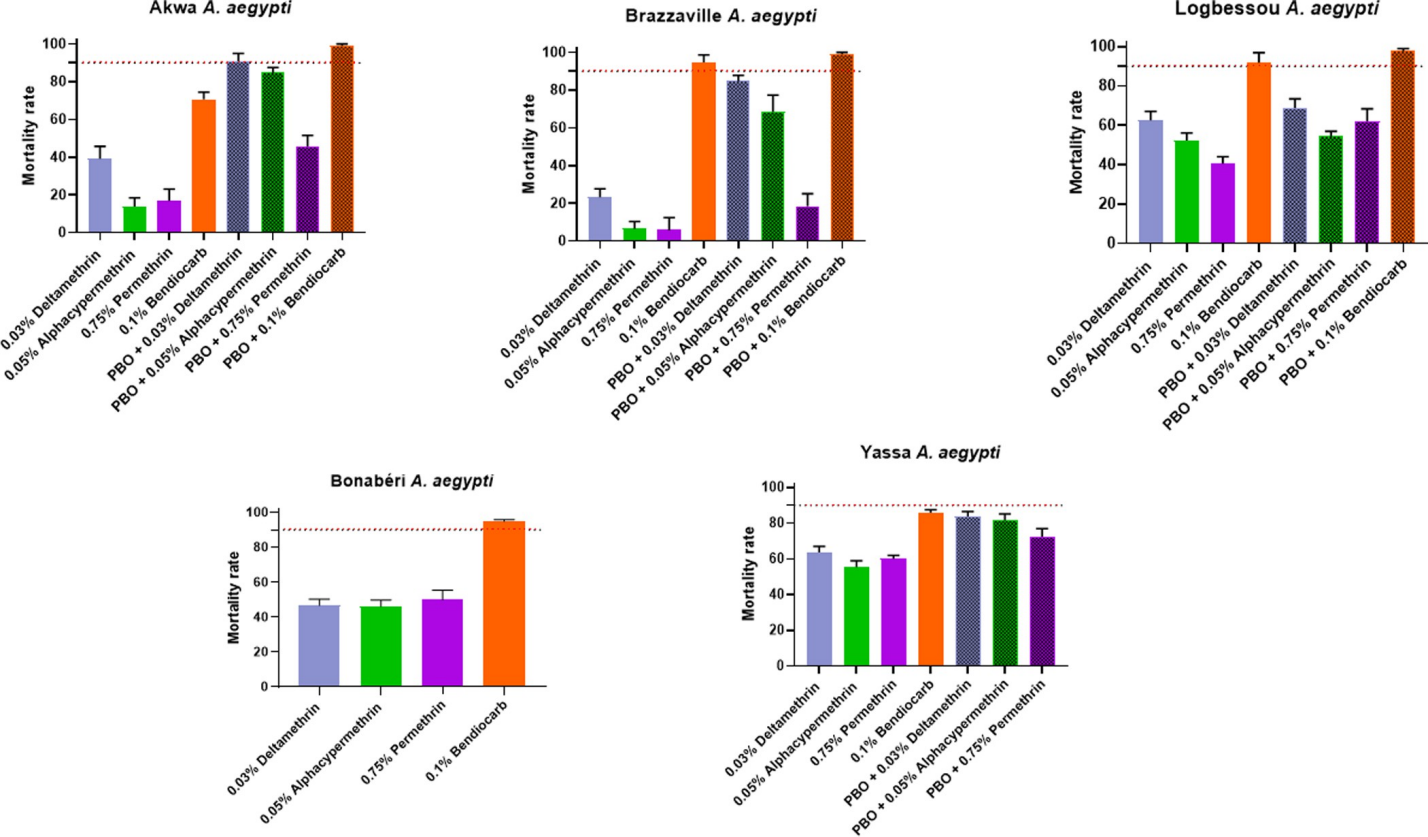
Mortality rates of adult *Aedes aegypti* from five locations (per environment) in Douala when exposed to insecticides alone or with 1 h pre-exposure to synergist. Error bars represent standard error of the mean. *PBO* Piperonyl butoxide.

All populations tested were resistant to three pyrethroids used with mortality rates varying from 23.30% (Brazzaville) to 63.74% (Yassa) to deltamethrin, from 6.57% (Brazzaville) to 55.45% (Yassa) to alphacypermethrin and from 5.83% (Brazzaville) to 60.16% (Yassa) to permethrin. With respect to bendiocarb, we observed resistance in Akwa and Yassa with mortality rates of 70.62% and 85.89% respectively. Probable resistance was observed in Brazzaville, Bonabéri and Logbessou with mortality rate between 91.79 and 94.91% (Fig 3).

#### Insecticide resistance profile of *A*. *albopictus* in Douala

**Fig 4:**
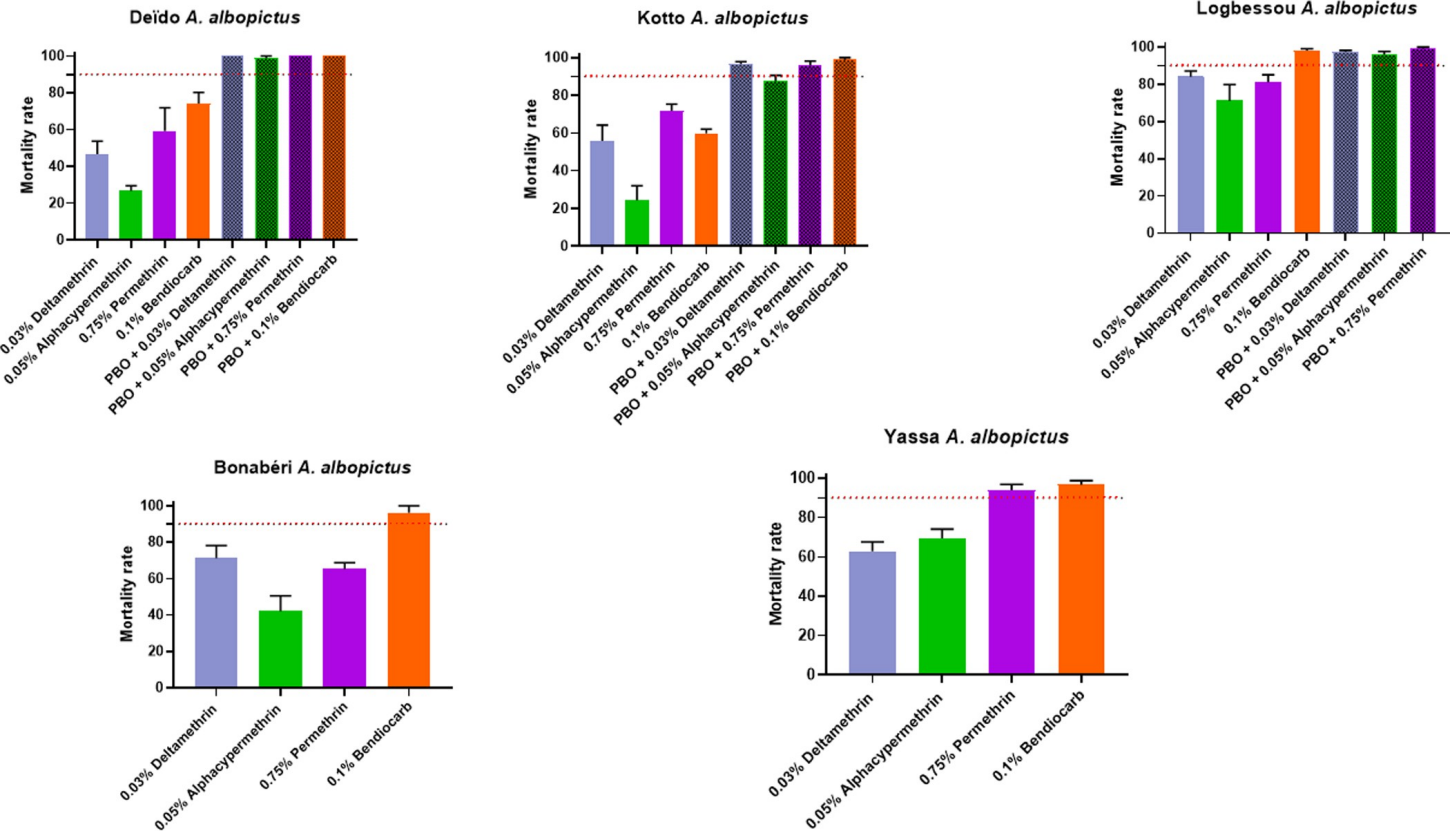
Mortality rates of adult *Aedes albopictus* from five locations (per environment) in Douala when exposed to insecticides alone or with 1 h pre-exposure to synergist. Error bars represent standard error of the mean. *PBO* Piperonyl butoxide.

All populations tested were resistant to all pyrethroids used with mortality rates varying from 6.72% (Deïdo) to 84.11% (Logbessou) to deltamethrin, from 24.35% (Kotto) to 71.21% (Logbessou) to alphacypermethrin, and from 59.26% (Deïdo) to 81.15% to permethrin. To bendiocarb, we observed susceptibility in Logbessou, probable resistance in Bonabéri and Yassa (mortality rate around 96%), confirmed resistance in Deïdo and Kotto (mortality rates of 74.17 and 59.78% respectively) (Fig 4).

*Tests with synergist PBO*. To evaluate if the resistance observed in some populations was due by the Cyt-P450 genes, mosquitoes were pre-exposed to synergist PBO. The analyses revealed a significant recovery of susceptibility to insecticide with pre-exposure to PBO in most populations tested for both species. This was the case of *A*. *aegypti* from Logbessou where a recovery of susceptibility was reported to deltamethrin (84.11± 3.09% of mortality without PBO pre-exposure vs 97.28± 0.90% of mortality after PBO pre-exposure, *P* < 0.05), permethrin (81.15 ± 3.97% of mortality without PBO pre-exposure vs 99,10 ± 0,89% of mortality after PBO pre-exposure, *P* < 0.05), and alphacypermethrin (71.21 ± 8.69% of mortality without PBO pre-exposure vs 95,94 ± 1,73% of mortality after PBO pre-exposure, *P* < 0.05).

**Fig 5:**
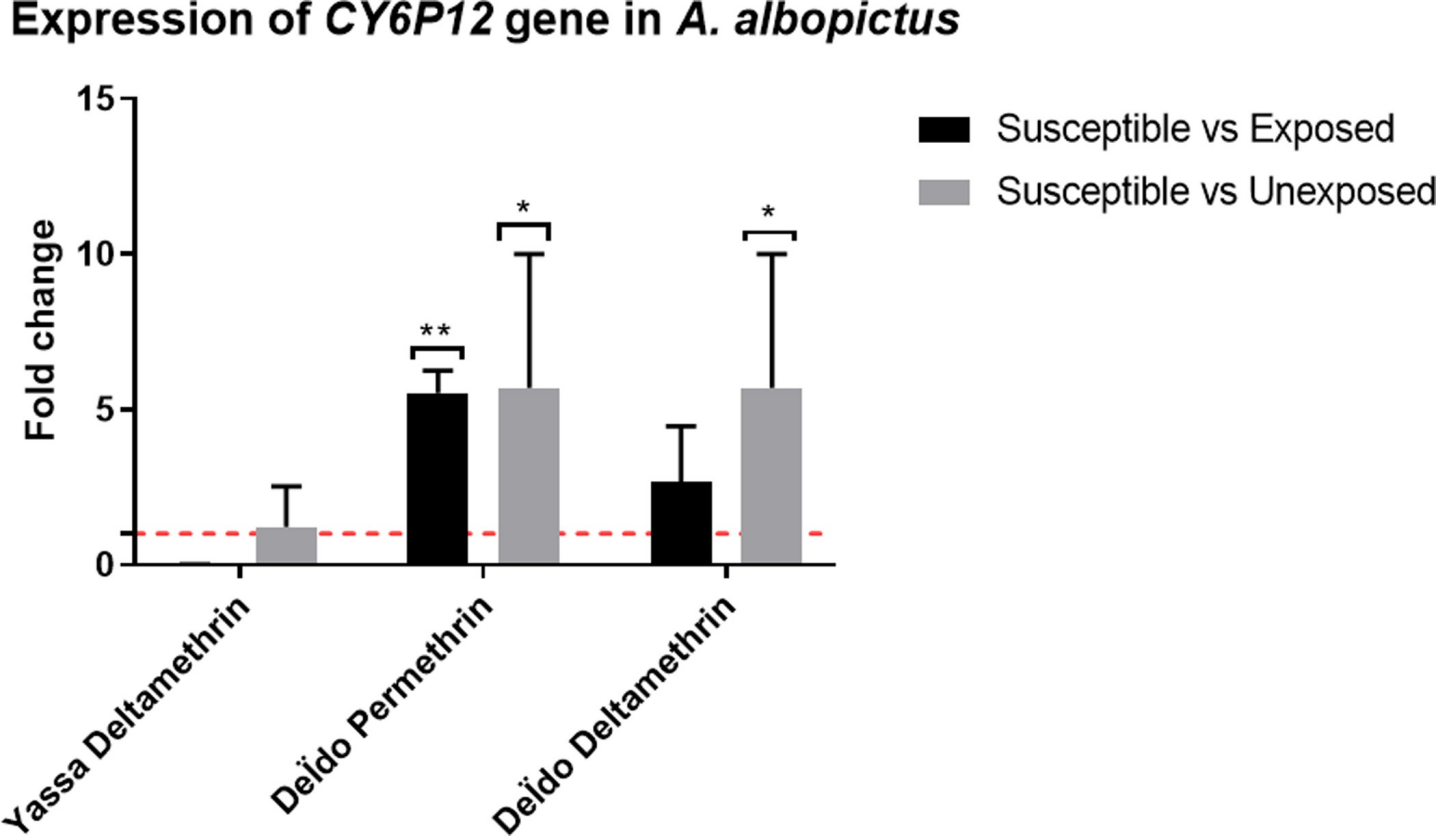
Expression of the *CYP6P12* gene in *A*. *albopictus* samples from Douala city.

*Expression of detoxification Genes*. The qPCR analyses revealed that the only gene (*Cyp6P12*) whose expression was assessed in *A*. *albopictus* populations was found to be significantly overexpressed in the permethrin resistant Deïdo samples compared to susceptible VCRU lab strain (FC = 5.54 ± 0.73, P = 0.001); whereas in the deltamethrin-resistant Deïdo samples and the alphacypermethrin-resistant Yassa samples, the expression was not significantly different (Fig 5).

**Fig 6:**
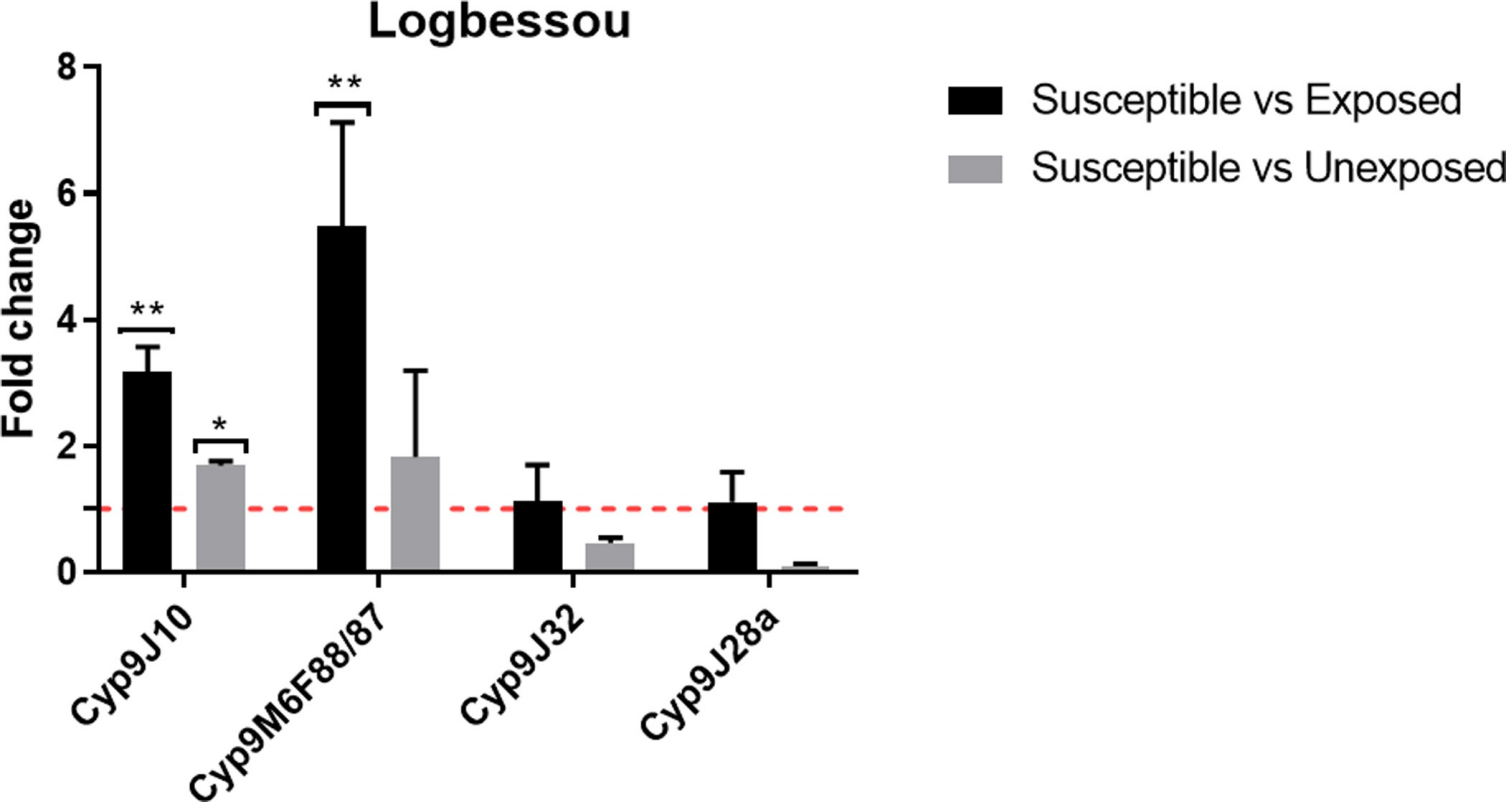
Expression of the *CYP9J10*, *CYP9M6F88/87*, *CYP9J32* and *CYP9J28a* genes in *A*. *aegypti* samples from Logbessou, Douala city.

**Fig 7:**
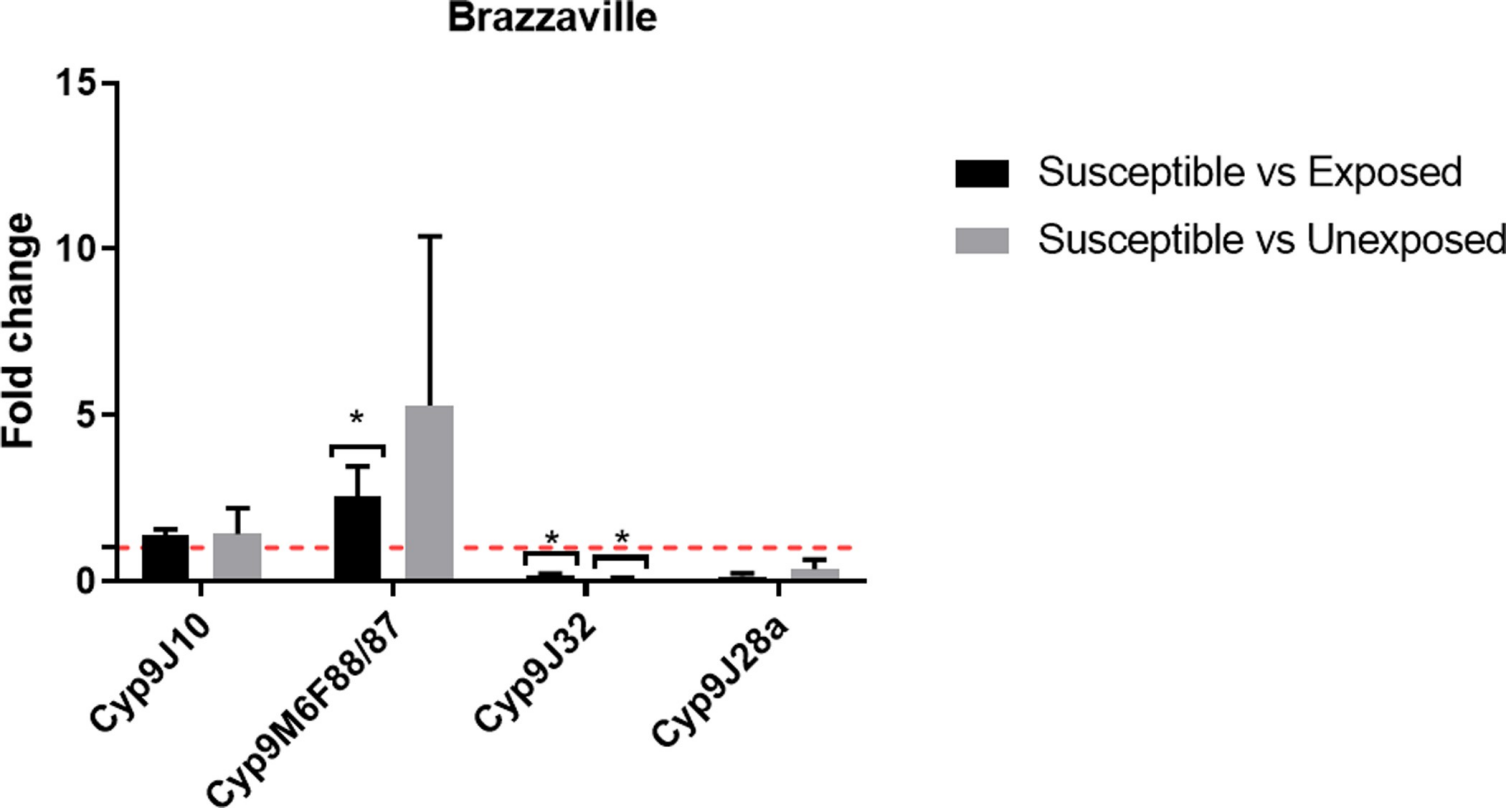
Expression of the *CYP9J10*, *CYP9M6F88/87*, *CYP9J32* and *CYP9J28a* genes in *A*. *aegypti* samples from Brazzaville, Douala city.

Two among the four Cyt-P450 genes assessed in *A*. *aegypti* were significantly overexpressed in field populations compared to susceptible Benin lab stain (Figs 6 and 7). *Cyp9M6F88/87* was overexpressed in Logbessou samples resistant to deltamethrin (FC = 5.49 ± 1.64, P = 0.003) and in Brazzaville samples resistant to permethrin (FC = 2.54 ± 0.90, P = 0.016); while *Cyp9J10* was overexpressed in Logbessou samples and resistant to deltamethrin (FC = 3.16 ± 0.40, P = 0.013).

*Knockdown resistance (kdr) genotyping*. Three *kdr* mutations were genotyped F1534C, V1016I and V410L in eight locations of Douala (Table 3).

**Table 3:** Genotypes of *Aedes aegypti* populations from Douala, Cameroon considering V1016I, V410L *kdr* and F1534C mutation in the VGSC.

qPCR results Location	VV/VV/FF	VV/VV/CC	VV/VV/FC	VI/VV/CC	VV/VL/CC	VV/VL/FC	VV/LL/FC	VV/LL/CC	VI/VV/FC	VI/VL/CC	VI/VL/FC	II/LL/CC	II/VL/CC	VI/LL/CC	n
Yassa	0.37	0.4	0.17	0	0	0	0	0	0	0.03	0.03	0	0	0	30
Bonaberi	0.04	0.31	0.1	0.04	0.03	0.03	0	0	0	0.38	0	0.07	0	0	29
Bepanda	0	0.23	0	0	0	0	0	0	0	0.33	0.07	0.34	0.03	0	30
Logbessou	0.04	0.57	0.13	0.03	0	0	0	0	0	0.23	0	0	0	0	30
Deïdo	0	0.15	0	0.11	0.15	0	0.04	0.07	0	0.3	0	0.04	0.07	0.07	27
Kotto	0.07	0.44	0	0.04	0	0	0	0	0	0.34	0.04	0.07	0	0	27
Brazzaville	0	0.69	0.08	0	0	0	0	0	0	0.19	0.04	0	0	0	26
Akwa	0.04	0.5	0.08	0	0.04	0	0	0	0.03	0.23	0	0.08	0	0	26

n: Number of samples tested; qPCR results (1016+410+1534); V: Valine; I: Isoleucine; L: Leucine; F: Phenylalanine; C: Cysteine.

Concerning V1016I *kdr* genotyping, a total of 230 samples were examined. Among these, 131 (56.96%) were homozygote wild type (1016V/V), 76 (33.04%) were heterozygote (1016V/I) and 23 (10%) were homozygote mutant (1016I/I). Overall, allelic frequency of homozygote wild type was 73.48% while for homozygote mutant was 26.52% (S1 Fig).

For V410L *kdr* genotyping, we examined around 226 samples of which 125 (55.31%) were homozygote wild type (410 V/V), 78 (34.51%) were heterozygote (410V/I) and 23 (10.18%) were homozygote mutant (410I/I). Overall, allelic frequency of homozygote wild type was 72.57% while for homozygote mutant was 27.43% (S2 Fig).

A total of 238 specimens were examined for F1534C genotyping. Overall allelic frequencies were 12.61% for homozygote wild type and 87.39% for homozygote mutant (S3 Fig). Analysis revealed that 16 (6.72%) were homozygote wild type (1534F/F), 28 (11.77%) were heterozygote (1534F/C) and 194 (81.51%) were homozygote mutant (1534C/C).

**Fig 8:**
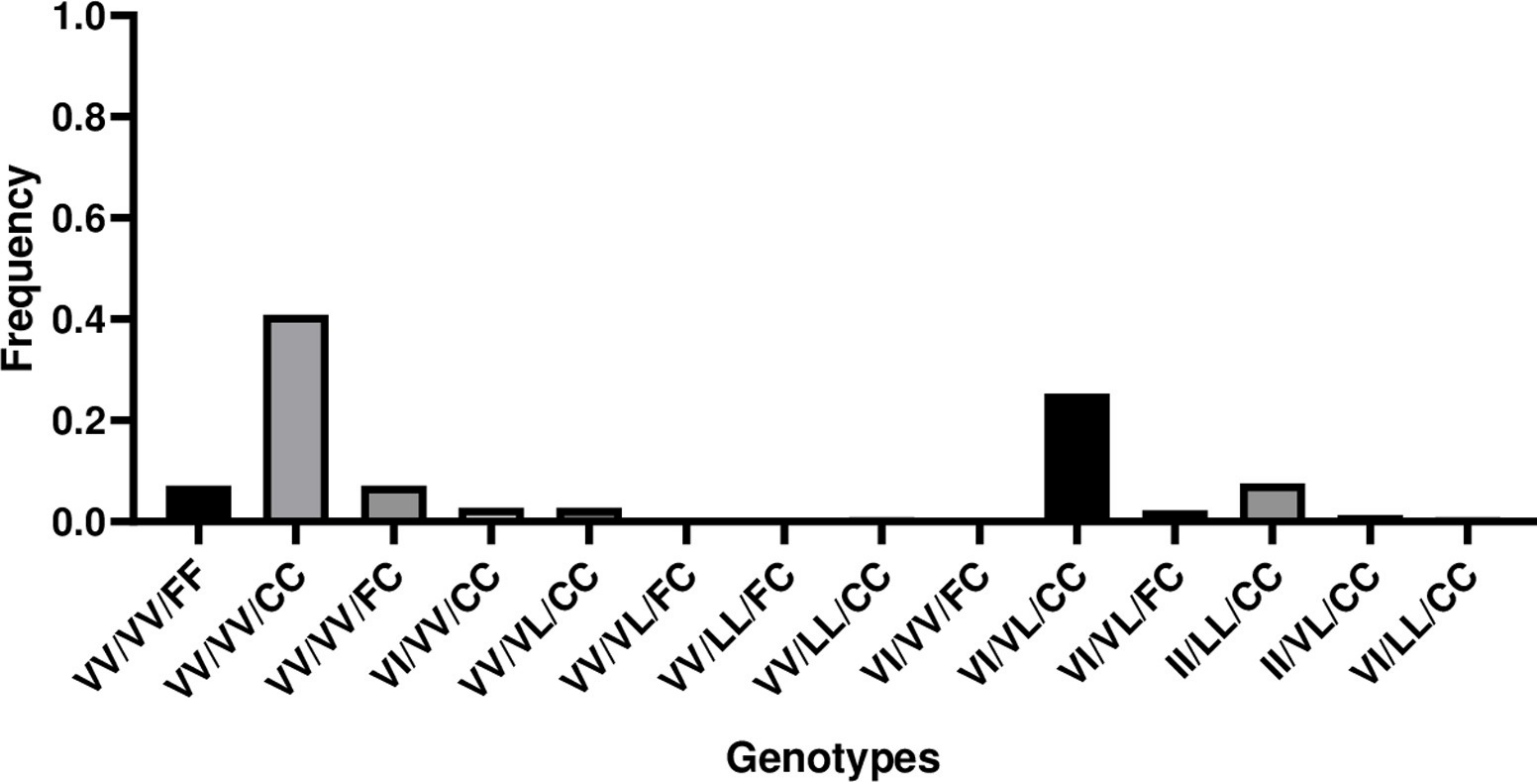
Frequency of *kdr* genotypes in *Aedes aegypti* mosquito from Douala, Cameroon considering F1534C, V1016I and V410L *kdr* mutation in the *VGSC*.

By considering the three *kdr* together (1016+410+1534), 225 samples of *A*. *aegypti* were analyzed and we found 14 genotypes: VV/VV/FF, VV/VV/CC, VV/VV/FC, VI/VV/CC, VV/VL/CC, VV/VL/FC, VV/LL/FC, VV/LL/CC, VI/VV/FC, VI/VL/CC, VI/VL/FC, II/LL/CC, II/VL/CC and VI/LL/CC (Fig 8). The frequency of each genotype varies according to the location. The only one susceptible genotype VV/VV/FF was present in five locations of the eight locations tested. The high frequency of this genotype was 37% (Yassa samples) followed by 7% (Kotto samples). The most predominant genotype VV/VV/CC was found in all the eight locations analyzed with frequency between 15 and 69% in Deïdo and Brazzaville respectively. Overall, the frequency of this genotype was 40,88% within the Douala *A*. *aegypti* population. The second dominant genotype was VI/VL/CC present in all the eight locations with a frequency between 3% and 38%. The frequency of this genotype in the population was 25.33%. We noticed that some genotypes were present in only five locations (VV/VV/FC and II/LL/CC), four locations (VI/VV/CC and VI/VL/FC), three locations (VV/VL/CC), two locations (II/VL/CC) or in only one location (VV/VL/FC, VV/LL/FC, VV/LL/CC, VI/VV/FC and VI/LL/CC).

## Discussion

This study aimed to assess the spatial distribution, the insecticide resistance profile of *A*. *aegypti* and *A*. *albopictus* in Douala and to explore the potential resistance mechanisms involved.

### Spatial distribution of *A*. *aegypti* and *A*. *albopictus* in Douala

Results showed that both species are present in all prospected neighbourhoods. *Aedes aegypti* was the predominant species in all neighbourhoods located in downtown while *A*. *albopictus* was more predominant in suburban localities. Previous studies reported the same observation in the city of Douala [5,6].

The predominance of *A*. *aegypti* in the downtown is consistent with previous works on the spatial distribution of *A*. *aegypti* and *A*. *albopictus* in other cities in Central Africa [8,10], in Asia [37] and in South America [38]. In these localities, it was clearly demonstrated that *A*. *aegypti* preferentially colonises man-made containers located in areas with high building density [6,37,39], whereas *A*. *albopictus* preferentially colonised breeding sites surrounded by vegetation [5,6,8]. In this study the predominant breeding sites were used tyres, which are described in previous studies as preferential breeding sites for *Aedes* [8] because they offer not only very good shelter from predators but also from heat. The suburban neighbourhoods (Kotto, Logbessou, Yassa) which have a high vegetation cover compared to the downtown neighbourhoods (Deïdo, Akwa, Brazzaville and Bépanda) therefore offer a more favourable environment for the development of *A*. *albopictus*. This is consistent with previous studies conducted in some suburban neighbourhoods of Douala showing that *A*. *albopictus* is the predominant species in this environment [40]. Overall, analyses revealed that *A*. *aegypti* is the predominant species in the city of Douala, which corroborates previous observations [5,6]. This suggests that the environmental factors in Douala are more favourable to the development of this species in contrast to what is generally observed in localities in the southern part of Cameroon located below 6°N [5].

### Insecticide resistance profile of *A*. *aegypti* and *A*. *albopictus*

This study revealed that both species *A*. *aegypti* and *A*. *albopictus* are resistant to all insecticides tested (deltamethrin, alphacypermethrin, permethrin and bendiocarb). Indeed, resistance to permethrin 0.75% and deltamethrin 0.03% had already been reported in *A*. *aegypti* in Douala [23], in other cities in Cameroon [9,23], and other regions of the world such as Saudi Arabia [41] and Burkina Faso [42]. The resistance to 0.03% deltamethrin and 0.75% permethrin observed in *A*. *albopictus* corroborates with those previously obtained in Congo [43], in Vietnam, in Singapore and in Italy [44] for permethrin and in Yaoundé for deltamethrin [9].

Globally, *A*. *aegypti* is more resistant to pyrethroids than *A*. *albopictus*. Similar results were obtained in other parts of the world including Malaysia [45]. However, these observations are different from those reported in the city of Yaoundé (Cameroon), indicating that the level of resistance observed in *A*. *albopictus* to pyrethroids was higher than in *A*. *aegy*pti [9]. The resistance of *A*. *aegypti* and *A*. *albopictus* to pyrethroids could pose a serious threat to vector control programs, because pyrethroids are the main class of insecticide recommended for the control of adult *Aedes* mosquitoes notably in case of outbreaks [11].

A loss of susceptibility to bendiocarb was observed for both *Aedes* species. Similar patterns have been observed previously in Saudi Arabia [41], in Burkina Faso [42], Pakistan [46] and Malaysia [45].

Resistance or loss of susceptibility observed to these insecticides (deltamethrin, permethrin and bendiocarb) in both *Aedes* species is difficult to explain because in Central Africa, specific insecticide treatments targeting *Aedes* spp. are currently very limited or non-existent [9,47]. This observation raises the question of the origin of the selection pressure by these species. As suggested previously [9,47], domestic use of insecticides through Indoor Residual Spraying (IRS) and impregnating bed nets, mass distribution of long-lasting insecticidal nets (LLINs) by national malaria control programme, and agriculture use could be the main source of resistance selection in *Aedes* vectors in Central Africa. Indeed, the use of pesticides in agriculture for the protection of market gardening could also promoted the emergence of resistance in mosquitoes by contamination of breeding sites and resting places of mosquitoes.

### Resistance mechanism involved

Synergist assay with pre-exposure to PBO showed a partial or full recovery of susceptibility to all insecticides used in *A*. *aegypti* and *A*. *albopictus*. These observations are consistent with previous studies in Africa [9,18,23,43,48] and abroad [14,15]. This result indicates that the cytochrome P450 monooxygenases are playing the main role in the resistance of deltamethrin, alphacypermethrin, permethrin and bendiocarb. This result is supported by the overexpression of some genes analysed such as *CYP6P12* in Deïdo in *A*. *albopictus* samples resistant to permethrin or *Cyp9M6F88/87* in Logbessou and Brazzaville in *A*. *aegypti* samples resistant to deltamethrin and permethrin respectively. The *CYP6* and *CYP9* sub-families, were previously found to be associate with resistance to pyrethroids [14,15].

In this study three *kdr* mutations F1534C, V1016I and V410L known as involved in pyrethroids resistance in *Aedes* mosquito are reported. These mutations have already been reported in Cameroon [23–25] and other countries in Africa [18,42,49–52] and abroad [22].

The F1534C mutation is common in *A*. *aegypti* and has a cosmopolitan distribution [12]. This mutation has been reported in West Africa including Ghana [21] and Burkina Faso [18,42]. Although not previously reported in Central Africa, previous work of Yougang et *al*. reported for the first time the existence of this mutation in *A*. *aegypti* in two cities in Cameroon [23]. The results of this work confirmed the presence of this *kdr* mutation in the city of Douala with a very high allelic frequency (87.39%). This high allelic frequency is similar to previous one reported in Douala [24]. The occurrence of these mutations is likely to impact the effectiveness of pyrethroid-based control measures against *A*. *aegypti* currently implemented in some African countries [51]. The combination of two of these mutations or all three mutations contribute strongly to increase resistance in *A*. *aegypti*. For example, V410L, alone confers low levels of resistance but co-evolved with V1016I or F1534C yielding higher levels of resistance [18,22,51]. Considering the tree *kdr* mutations together 14 genotypes were found, among them two were the most abundant: VV + VV + CC and VI + VL + CC (1016+410+1534). Similar observation was reported in Brazil [53]. Further studies are needed to establish whether there is an association between these mutations and phenotypic resistance in *A*. *aegypti* population from Cameroon.

## Conclusion

This study revealed that *A*. *aegypti* and *A*. *albopictus* are present in city of Douala with *A*. *aegypti* as the most abundant. Both *Aedes* species are resistance to all insecticides tested and three *kdr* mutations have been detected in *A*. *aegypti* samples. A full or partial recovery of susceptibility observed after pre-exposure of mosquitoes to PBO, suggests a major role of P450 genes especially *CYP6P12* and *Cyp9M6F88/87* overexpressed in resistant mosquitoes. These findings are important for the control of *Aedes* mosquitoes in the city of Douala, but more studies need to be conducted to obtain additional information on other insecticide resistance mechanisms such as metabolic resistance.

## Supporting information

S1 Fig Distribution of V1016 and 1016I alleles in *A*. *aegypti* population in Douala city. QGIS version 3.14.16, was used to generate the map using open access share files (https://gadm.org/).
(TIF)

S2 Fig Distribution of V410 and 410L alleles in *A*. *aegypti* population in Douala city. QGIS version 3.14.16, was used to generate the map using open access share files (https://gadm.org/).
(TIF)

S3 Fig Distribution of F1534 and 1534C alleles in *A*. *aegypti* population in Douala city. QGIS version 3.14.16, was used to generate the map using open access share files (https://gadm.org/).
(TIF)

S1 Table qPCR raw data for gene expression. (XLSX)
