# Supplementary figures and images for: Spatial distribution and insecticide resistance profile of Aedes aegypti and Aedes albopictus in Douala, the most important city of Cameroon
Source: PLoS One. 2022 Dec 13;17(12):e0278779. doi: 10.1371/journal.pone.0278779 (PMC9746985; doi:10.1371/journal.pone.0278779)

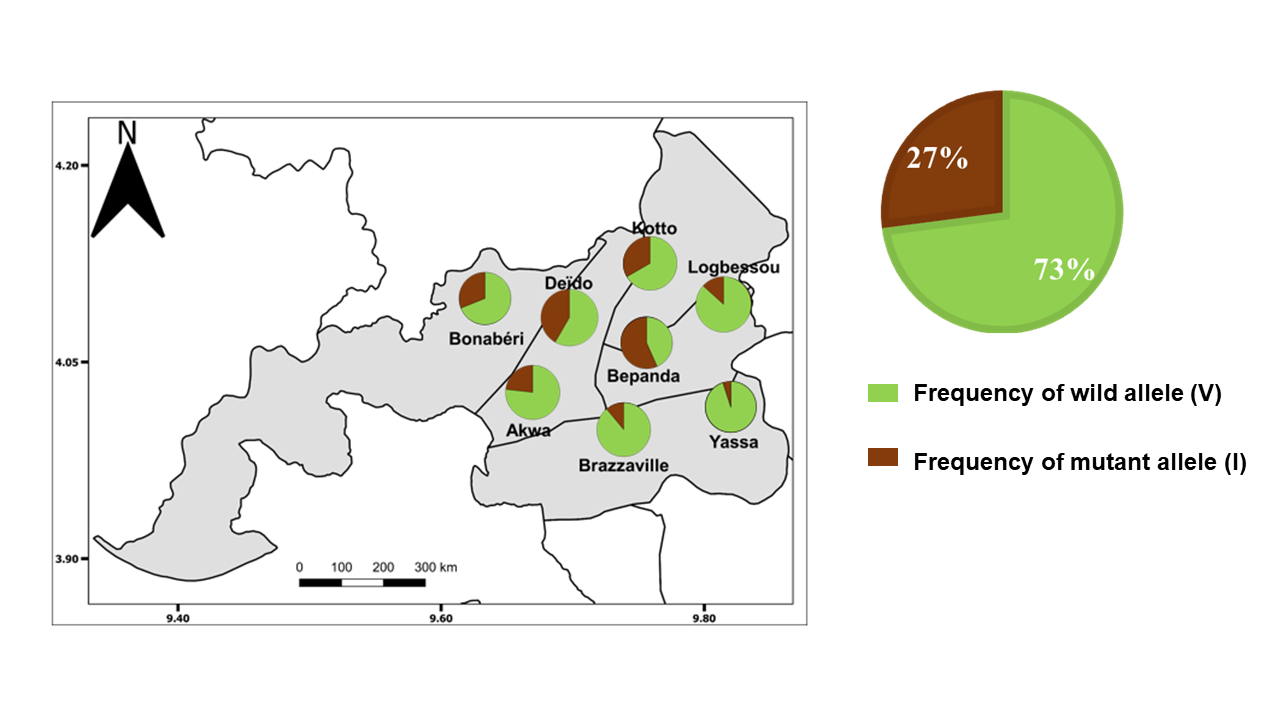

Supplement: S1 Fig — QGIS version 3.14.16, was used to generate the map using open access share files (https://gadm.org/). (TIF) [file pone.0278779.s001.tif]

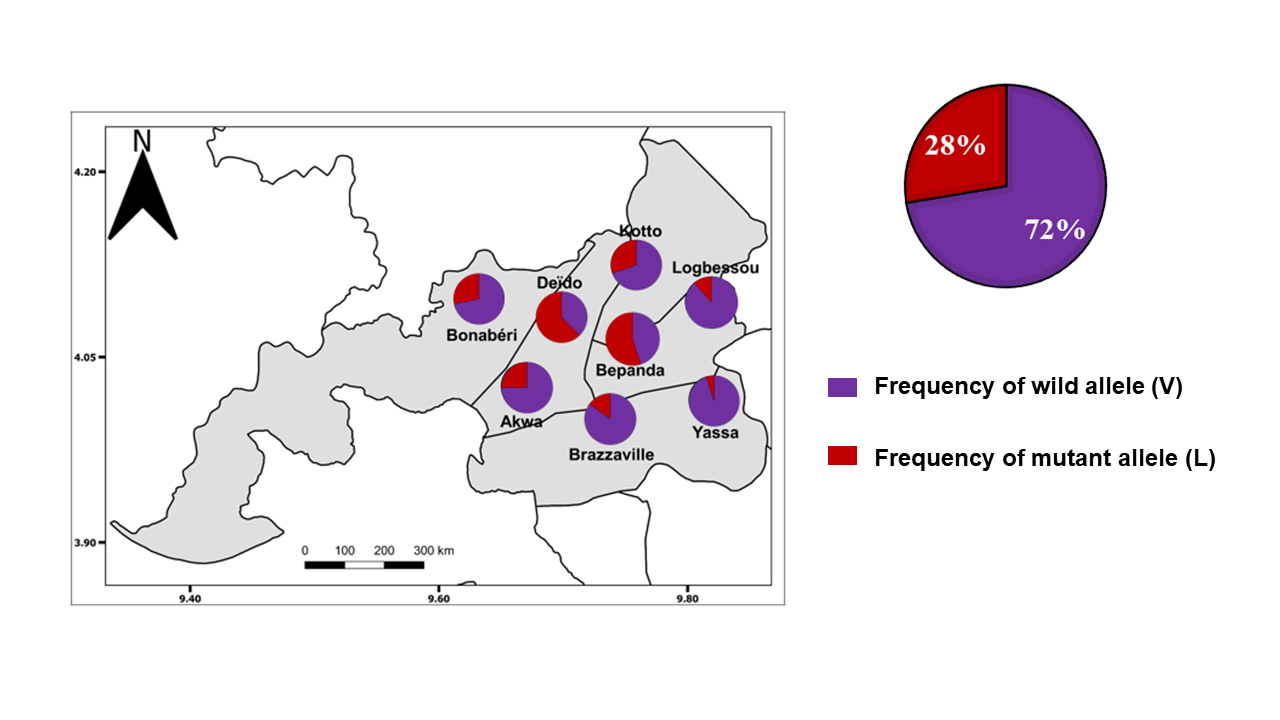

Supplement: S2 Fig — QGIS version 3.14.16, was used to generate the map using open access share files (https://gadm.org/). (TIF) [file pone.0278779.s002.tif]

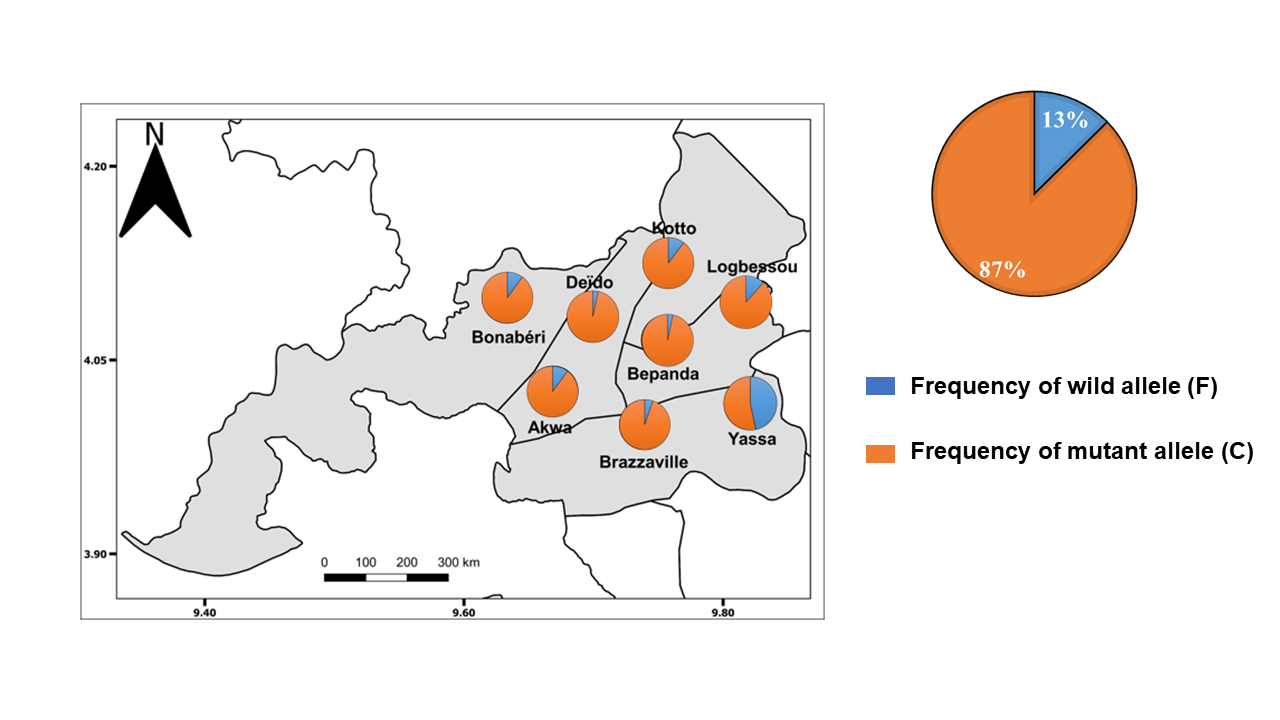

Supplement: S3 Fig — QGIS version 3.14.16, was used to generate the map using open access share files (https://gadm.org/). (TIF) [file pone.0278779.s003.tif]
